# An immune relevant signature for predicting prognoses and immunotherapeutic responses in patients with muscle‐invasive bladder cancer (MIBC)

**DOI:** 10.1002/cam4.2942

**Published:** 2020-02-25

**Authors:** Wen Jiang, Dandan Zhu, Chenghe Wang, Yu Zhu

**Affiliations:** ^1^ Department of Urology Ruijin Hospital Shanghai Jiao Tong University School of Medicine Shanghai China

**Keywords:** gene expression, immune infiltration, immunotherapeutic response, muscle‐invasive bladder cancer (MIBC), prognosis

## Abstract

Immune checkpoint inhibitors (ICIs) are novel treatments that significantly improve the survival time of MIBC patients, but immunotherapeutic responses are different among MIBC patients. Therefore, it is urgent to find predictive biomarkers that can accurately identify MIBC patients who are sensitive to ICIs. In this study, we computed the relative abundances of 24 immune cells based on the expression profiles of MIBC patients using single‐sample gene set enrichment analysis (ssGSEA). Unsupervised clustering analysis of the 24 immune cells was performed to classify MIBC patients into different immune‐infiltrating groups. Genome (gene mutation and copy number variation), transcriptome (mRNA, lncRNA, and miRNA), and functional enrichment were found to be heterogeneous among different immune‐infiltrating groups. We identified 282 differentially expressed genes (DEGs) associated with immune infiltration by comparing the expression profiles of patients with different immune infiltration profiles, and 20 core prognostic DEGs were identified by univariate Cox regression analysis. An immune‐relevant gene signature (TIM signature) consisting of nine key prognostic DEGs (CCDC80, CD3D, CIITA, FN1, GBP4, GNLY, SPINK1, UBD, and VIM) was constructed using least absolute shrinkage and selection operator (LASSO) Cox regression analysis. Receiver operating characteristic (ROC) curves and subgroup analysis confirmed that the TIM signature was an ideal biomarker for predicting the prognosis of MIBC patients. Its value in predicting immunotherapeutic responses was also validated in The Cancer Genome Atlas (TCGA) cohort (AUC = 0.69, 95% CI = 0.63‐0.74) and the IMvigor210 cohort (AUC = 0.64, 95% = 0.55‐0.74). The TIM signature demonstrates a powerful ability to distinguish MIBC patients with different prognoses and immunotherapeutic responses, but more prospective studies are needed to assess its reliability in the future.

## Graphical abstract

Immune checkpoint inhibitors (ICIs) are novel treatments for MIBC and significantly improve survival time of MIBC patients, but ICIs are only effective for minority of MIBC patients. We first constructed a tumor immune infiltration–associated gene signature (TIM signature) for MIBC patients to predict prognosis and immunotherapeutic response by comprehensively comparing expression profiles of MIBC patients with different immune infiltrations in this study. This signature might be useful for future clinical immunotherapy of MIBC patients.

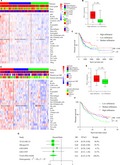

## 1 INTRODUCTION

Bladder cancer (BC) is a common malignancy of the urinary system, with an estimated 4 30 000 new cases diagnosed worldwide per year.^1^ Approximately 25% of BC patients are diagnosed with muscle‐invasive bladder cancer (MIBC), which in the past 30 years still has poor prognosis and lacks effective therapeutic options.^2^ Traditionally, platinum‐based chemotherapy was considered the first‐line regimens for advanced BC, but its antitumor ability was limited by drug toxicities, making half of BC patients ineligible for chemotherapy.^3^ Since 1976, a well‐known immunotherapy, Bacillus Calmette‐Guérin (BCG), has been used as the gold standard treatment for nonmuscle‐invasive bladder (NMIBC) patients who are at a high risk of progression,^4^, ^5^ and immunotherapy has been suggested for the treatment of BC. For MIBC, the inhibition of immune checkpoints such as programmed cell death ligand 1 (PD‐L1)/programmed cell death protein 1 (PD‐1) can reactivate immune cells cytotoxicity and cause tumor regression.^6^ Additionally, several immune checkpoint inhibitors (ICIs) such as atezolizumab (PD‐L1 inhibitor) and nivolumab (PD‐1 inhibitor) were approved by the Food and Drug Administration (FDA) for curing advanced MIBC, and they significantly prolong the survival time of advanced MIBC patients.^7^, ^8^

However, the immunotherapeutic responses to ICIs are variable among MIBC patients, with some patients achieving complete remission and others showing continuous progression, and the cost of ICI treatments is still high for the average family.^9^ Therefore, predictive biomarkers that can accurately identify patients who are sensitive to ICIs are urgently needed. To discriminate immunotherapeutic responders from nonresponders, several useful biomarkers such as PD‐L1 expression, tumor mutational burden (TMB), tumor‐infiltrating phenotypes, and the microbiome pattern of patients have been identified.^10^ However, some of these biomarkers still lack enough stability for the prediction of immunotherapeutic responses. For example, PD‐L1 expression and tumor‐infiltrating immune cells are estimated by evaluating the staining density and area of immunohistochemical sections, and thus these biomarkers cannot be precisely quantified, and are subject to interobserver variation, especially at low expression levels.^11^ Meanwhile, some biomarkers have not been validated in MIBC patients; therefore, additional biomarkers that can accurately predict the immunotherapeutic responses of MIBC patients before ICI treatment are still needed.

Several studies have reported that the effectiveness of immunotherapies depends on reactivating the host immune response of the tumor immune microenvironment (TME).^12^, ^13^ However, the TME, which comprises immune cells, vessel cells, fibroblasts, and the extracellular matrix, is diverse across tumor patients, and different TME patterns can drive wither tumor repression or progression.^14^ As described in a previous study, patients with a “hot” or highly infiltrated TME have more preexisting immune reserves, suggesting that ICI‐based monotherapy will likely be effective for these patients, but patients with a “cold” or non‐infiltrated TME always lack preexisting immune reserves, indicating that ICIs alone will not be sufficient.^12^ Therefore, an adequate assessment of MIBC patients TME will be helpful for predicting immunotherapeutic responses and formulating appropriate treatment protocols. Different from conventional immunohistochemical staining, computer algorithms based on transcriptome data are increasingly being used to determine the TME patterns of tumor patients,^15^ and these methods have been shown to be very reliable and not susceptible to pathological subjective interference.^16^, ^17^

In this study, we systematically analyzed the immune landscapes of four MIBC cohorts based on transcriptome data, and then the genomics differences of patients with different levels of immune infiltration were compared. Meanwhile, immune relevant genes were identified by comparing the transcriptomic data of MIBC patients with different infiltration patterns. Finally, a useful gene signature named the TIM signature was developed using a least absolute shrinkage and selection operator (LASSO) Cox regression model, and this signature was found to be highly associated with immune infiltration in MIBC patients and robust for predicting the prognoses and immunotherapeutic responses of MIBC patients after cross‐validation with other cohorts.

## 2 MATERIALS AND METHODS

### 2.1 Data acquisition and processing

Four MIBC cohorts (TCGA‐BLCA, IMvigor210, http://www.ncbi.nlm.nih.gov/geo/query/acc.cgi?acc=GSE13507, and http://www.ncbi.nlm.nih.gov/geo/query/acc.cgi?acc=GSE32894), which had detailed follow‐up information, were included in this study by searching The Cancer Genome Atlas (TCGA, https://tcga-data.nci.nih.gov/) and Gene Expression Omnibus (GEO, http://www.ncbi.nlm.nih.gov/geo/). For the TCGA‐BLCA cohort, high‐throughput data, including RNA‐sequencing data (fragments per kilobase of exon per million reads mapped (FPKM)), miRNA‐sequencing data, DNA copy number data, and mutation profiles of MIBC samples with detailed clinicopathological information were downloaded, and a total of 385 MIBC patients (T2, T3, and T4) were finally included in this study. To standardize the data, RPKM values were transformed into transcripts per kilobase million (TPM) values, and the relative expression level of each gene was finally presented in the form of log2(TPM + 1) to narrow the large numeric span. Ensembl IDs were transformed into gene symbols and biotypes referred to the GENCODE project gene annotation file (version 22, GRCh38). Then, mRNAs and lncRNAs were separated according to the annotated biotypes. For the IMvigor210 cohort, standardized RNA‐sequencing data of 195 MIBC patients with corresponding clinicopathological data were extracted from the IMvigor210CoreBiologies R package.^18^ After searching GEO, the microarray data of two MIBC cohorts (http://www.ncbi.nlm.nih.gov/geo/query/acc.cgi?acc=GSE13507 (n = 62) and http://www.ncbi.nlm.nih.gov/geo/query/acc.cgi?acc=GSE32894 (n = 93)), which had detailed survival data, were downloaded.^19^, ^20^

### 2.2 Abundance inference of immune cells from transcriptomics data

Gene markers of 24 immune cells (activated DCs (aDCs), B cells, CD8 T cells, cytotoxic cells, DCs, eosinophils, immature DCs (iDCs), macrophages, mast cells, neutrophils, NK cells, NK‐CD56 bright cells, NK‐CD56 dim cells, plasmacytoid DCs (pDCs), T cells, T helper cells, T central memory (Tcm) cells, T effector memory (Tem) cells, T follicular helper (TFH) cells, T gamma delta (Tgd) cells, T helper 1 (Th1) cells, Th17 cells, Th2 cells, and regulatory T (Treg) cells) were obtained from a previous study.^21^ By using the gene markers, single‐sample gene set enrichment analysis (ssGSEA) was employed to infer the relative abundances of the 24 immune cells based on the expression profiling data.^15^, ^17^, ^22^, ^23^ Unsupervised clustering was performed to divide MIBC patients into different infiltrating groups according to the infiltrating densities of immune cells.

### 2.3 Identification of differentially expressed genes (DEGs), lncRNAs, and miRNAs

To identify genes associated with immune infiltration, the limma R package was used to generate the *P* value and fold change (FC) for each gene, and genes with *P* value ≤.05 and |log2 FC| ≥ 1 were defined as DEGs.^24^ The overlapping DEGs among three infiltrating groups were determined via a Venn diagram, which was generated using an online tool (http://bioinformatics.psb.ugent.be/webtools/Venn/). The above procedures were then repeated to determine differentially expressed lncRNA‐ and miRNA‐associated immune infiltration.

### 2.4 Establishment of a tumor immune infiltration–associated gene signature (TIM signature)

Unsupervised clustering of DEGs was performed to classify patients into three gene subtypes (G1, G2, and G3). Then, univariate Cox regression analysis was performed using the survival R package to determine the relevant prognostic DEGs. To construct the TIM signature, DEGs with a *P* value ≤.01 were defined as the core prognostic genes, and then LASSO Cox regression was performed to screen key prognostic genes from the core prognostic genes using the *glmnet* R package.^25^ Then, the TIM risk score was calculated for each patient according to the following formula:

$$\text{TIM risk score}\;=\beta_{1}\times \exp rG_{1}+\;\beta_{2}\times \exp rG_{2}+\;\ldots \beta_{n}\times \exp rG_{n}$$

where exprG is the expression level of the key prognostic genes and β is the regression coefficient that was generated by the LASSO Cox regression model. Next, the formula was applied in three other cohorts (IMvigor210, http://www.ncbi.nlm.nih.gov/geo/query/acc.cgi?acc=GSE13507, and http://www.ncbi.nlm.nih.gov/geo/query/acc.cgi?acc=GSE32894) to validate the stability of the TIM signature.

### 2.5 Functional enrichment analysis

Gene Ontology (GO) pathway analysis of the DEGs from the four cohorts was performed using the *clusterProfiler* R package.^24^ Significant GO terms were defined as biological pathways with a *P* value ≤.01. The *GOSemSim* R package was employed to evaluate the similarity among the significant GO terms of the four cohorts referring to the annotation data GO.db, and similar GO terms among the four cohorts were shown in the form of a heatmap and tree diagram.^26^ The enrichment scores of 50 classic biological pathways for MIBC samples were generated using the *GSVA* R package, and different biological pathways between the high‐infiltrating group and the low‐infiltrating group were identified by the *limma* R package. The median TIM score was used as a cutoff value to classify patients into the high‐risk score group and the low‐risk score group. Then, gene set enrichment analysis (GSEA) was performed to test whether genes in the high‐risk score group or low‐risk score group were enriched in the predefined Hallmark gene sets (v6.2, downloaded from http://software.broadinstitute.org/gsea/downloads.jsp) with the GSEA 3.0 application under the JAVA platform. After 1000 permutations, gene sets with values of *P* ≤.05 and values of false discovery rate (FDR) ≤0.05 were considered significant.

### 2.6 Immunotherapeutic response prediction

As mentioned before, the TME exerts a significant influence on the immunotherapeutic response of tumor patients.^27^ To explore the relationship between the TIM signature and the immunotherapeutic response, two computational methods were adopted to infer the immunotherapeutic response of TCGA‐BLCA patients. First, a web application named Tumor Immune Dysfunction and Exclusion (TIDE) (http://tide.dfci.harvard.edu) was used to infer the anti‐PD1 and anti‐CTLA4 immunotherapeutic response of each sample based on the transcriptome profiles of the TCGA‐BLCA cohort.^28^ Second, subclass mapping (https://cloud.genepattern.org/gp) was used to infer the immunotherapeutic response by measuring similarities between the transcriptome profiles of the TCGA‐BLCA cohort and that of 47 previous melanoma patients with detailed immunotherapeutic information.^29^, ^30^, ^31^ Finally, the TIM signature was fitted in the IMvigor210 cohort, which had detailed immunotherapeutic information, to validate its predictive ability of the immunotherapeutic response.

### 2.7 Statistical analysis

Differences between two groups were compared using unpaired Student's *t* test (normally distributed) or the Mann‐Whitney *U* test (non‐normally distributed). Differences among three groups or more were compared using the Kruskal‐Wallis test and one‐way analysis of variance. The oncoprint of the top 25 common mutated genes of MIBC patients was drawn using the ComplexHeatmap R package to depict the mutation landscapes of different subgroups.^32^ Amplified or deleted regions of the genome were identified using the Genomic Identification of Significant Targets in Cancer (GISTIC) 2.0 algorithm.^33^ Spearman or distance correlation analysis was used to compute correlation coefficients between every two factors. Survival differences among different groups were compared using Kaplan‐Meier curves followed by the log‐rank test. The chi‐square test or Fisher's exact test was used to analyze differences between rates of different groups. A fixed effects model (I^2^ ≤ 50%) or random effects model (I^2^ > 50%) was adopted using the meta R package to pool the HRs of multiple subgroups. To evaluate the diagnostic accuracy of the TIM signature, a receiver operating characteristic (ROC) curve was generated using the pROC R package to calculate AUC (area under the ROC curve) and 95% CI (confidence interval).

## 3 RESULTS

### 3.1 Immune‐infiltrating landscape of the TME in MIBC patients

**Figure 1:**
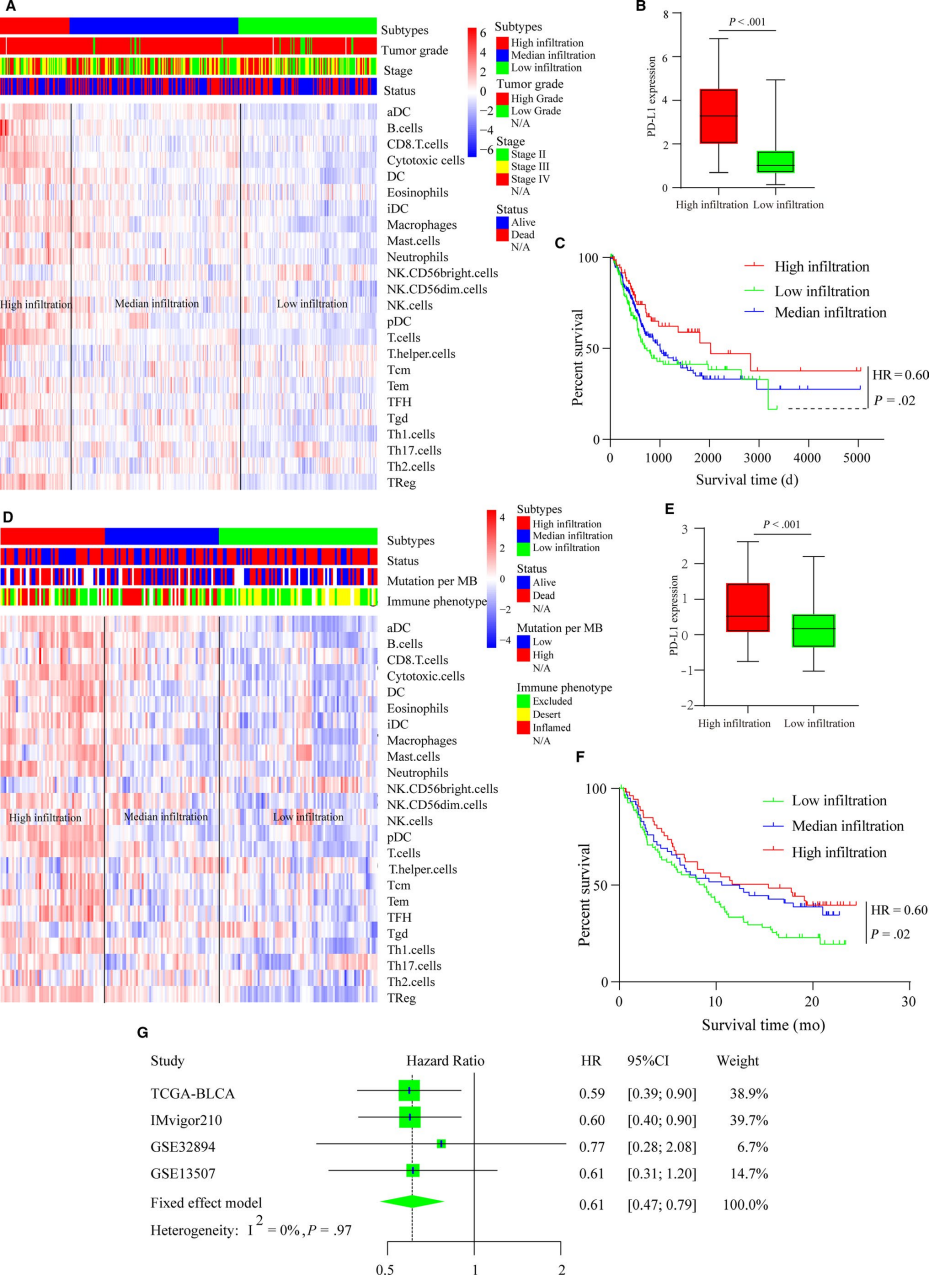
Immune‐infiltrating landscape of the TME in MIBC patients. (A) Unsupervised clustering of 24 immune cells for 384 MIBC patients from the TCGA‐BLCA cohort: high infiltration (red, n = 71), median infiltration (blue, n = 172), and low infiltration (green, n = 141). Parameters including tumor grade, pathological stage, and survival status are shown above the heatmap. (B) Expression level of PD‐L1 in the TCGA‐BLCA cohort patients with a high‐infiltrating TME (red) vs that in the patients with a low‐infiltrating TME (green). The bottom and top of the boxes represent the upper quartile and lower quartile percentiles, respectively. The whiskers encompass the maximum and minimum expression levels. The differences between the two groups were compared by the Mann‐Whitney U test (*P* < .001). (C) Kaplan‐Meier curves of the overall survival of the TCGA‐BLCA cohort patients with different infiltrating TME (high‐infiltrating TME vs low‐infiltrating TME, log‐rank test, *P* = .022). (D) Unsupervised clustering of 24 immune cells for 195 MIBC patients in the IMvigor210 cohort: high infiltration (red, n = 54), median infiltration (blue, n = 59), and low infiltration (green, n = 82). Parameters, including tumor grade, mutation load, and immune phenotype, are shown above the heatmap. (E) Expression level of PD‐L1 in patients in the IMvigor210 cohort with a high‐infiltrating TME (red) vs that in patients with a low‐infiltrating TME (green). The bottom and top of the boxes represent the upper quartile and the lower quartile percentiles, respectively. The whiskers encompass maximum and minimum expression levels. The differences between the two groups were compared by the Mann‐Whitney U test (*P* < .001). (F) Kaplan‐Meier curves of the overall survival of the IMvigor210 cohort patients with different infiltrating TME (high infiltrating TME vs low infiltrating TME, log‐rank test, *P* = .02). (G) Forest plot of the HRs for patients with high infiltration vs patients with low infiltration (pooled HR = 0.61, 95% CI = 0.47‐0.79; I^2^ = 0, *P* = .97)

The relative quantity of the 24 immune cells from the four cohorts (TCGA‐BLCA, IMvigor210, http://www.ncbi.nlm.nih.gov/geo/query/acc.cgi?acc=GSE13507, and http://www.ncbi.nlm.nih.gov/geo/query/acc.cgi?acc=GSE32894) was systematically estimated using the ssGSEA algorithm. The correlation between every two immune cells of the TCGA‐BLCA cohort is shown in Figure S1A, and we found several highly correlated couples of immune cells, such as T cell‐cytotoxic cells, macrophage‐Th1 cells, and B cell‐T cells. The pooled hazard ratios (HRs) of the 24 immune cells from the four cohorts are shown in Figure S1B. We found that high densities of CD8 T cells, cytotoxic cells, DCs, pDCs, T cells, T helper cells, TFH cells, and Tregs were associated with better prognosis in MIBC patients (HR < 1). Then, unsupervised hierarchical clustering of the 24 immune cells was performed to arrange patients with similar immune‐infiltrating patterns into one group. The sample sizes of the four cohorts were very different, with two >150 and two <100. To improve the stability of the results, we defined the number of immune‐infiltrating groups according to the respective sample size of the four cohorts. For the large sample cohorts (TCGA‐BLCA [n = 384] and IMvigor210 [n = 195]), we classified patients into three immune‐infiltrating groups: high infiltration, median infiltration, and low infiltration, and for the small sample cohorts (http://www.ncbi.nlm.nih.gov/geo/query/acc.cgi?acc=GSE13507 [n = 62] and http://www.ncbi.nlm.nih.gov/geo/query/acc.cgi?acc=GSE32894 [n = 93]), we classified patients into two immune‐infiltrating groups: high infiltration and low infiltration. The comprehensive immune landscapes of the four MIBC cohorts were depicted in the form of heatmaps (Figure 1A,D; Figure S2A,B). We found that there was significant heterogeneity among the immune‐infiltrating patterns of MIBC patients, and the result was highly consistent with a previous study.^34^ PD‐L1 mRNA expression levels were compared between the high‐infiltrating groups and the low‐infiltrating groups. We found that the mRNA expression levels of PD‐L1 were higher in the high‐infiltrating groups than in the low‐infiltrating groups (Figure 1B,E; Figure S2D) (Mann‐Whitney U test, *P* < .05). However, due to significant variation in the results (as evidenced by the large error bars), the results should be verified with additional samples in the future. The overall survival (OS) of the high‐infiltrating group was compared with that of the low‐infiltrating group, and we found that patients with a high immune‐infiltrating TME lived longer than patients with a low immune‐infiltrating TME (Figure 1C,F; Figure S2C). Finally, a fixed effects model was employed to pool the HRs of the four cohorts, and the result also validated that patients with a high‐infiltrating TME had longer OS times than patients with a low‐infiltrating TME (HR = 0.61, 95% CI = 0.47‐0.79; I^2^ = 0%, *P* = .97) (Figure 1G).

### 3.2 Genome and transcriptome characteristics of the different immune‐infiltrating subtypes

**Figure 2:**
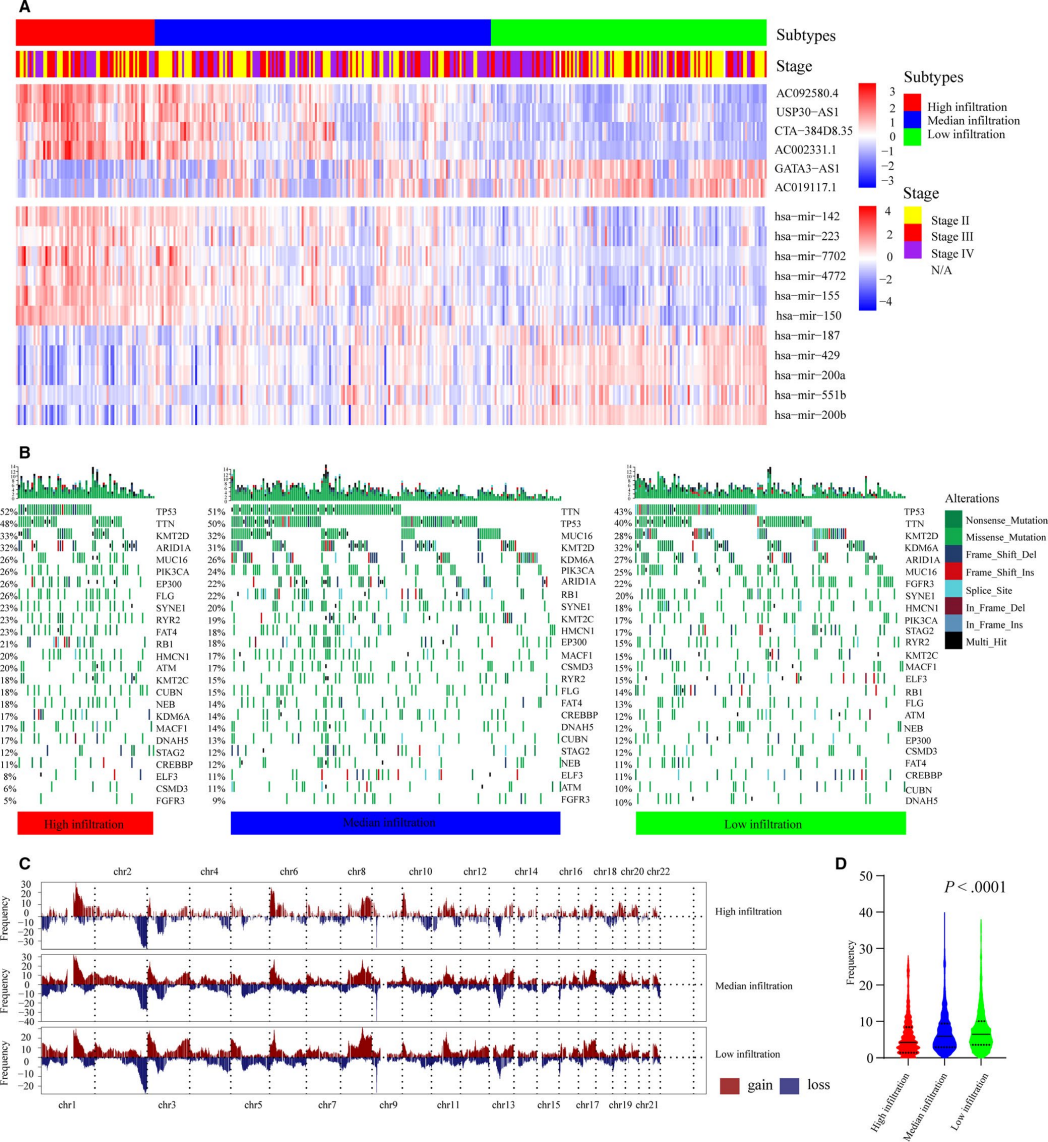
Genome and transcriptome characteristics of different immune‐infiltrating subtypes. (A) Heatmap of six differentially expressed lncRNAs and 11 differentially expressed miRNAs. Parameters including immune‐infiltrating subtypes and pathological stage are shown above the heatmaps. (B) Heatmap of 25 common mutated genes of patients from the TCGA‐BLCA cohort: high infiltration (red), median infiltration (blue), and low infiltration (green). The mutation frequencies of each patient are shown above the heatmap in the form of a bar graph. Annotations of nonsense mutations, missense mutations, frame‐shift deletions, frame‐shift insertions, splice sites, in‐frame deletions, in frame insertions, and multiple hits are shown on the right of the heatmap. (C) Gain (brown) or loss (blue) frequencies of copy number variations (CNVs) in the autosomes of MIBC patients from the TCGA‐BLCA cohort. (D) The frequencies of CNVs in the autosomes of MIBC patients were compared using the Kruskal‐Wallis test (*P* < .0001). The solid line represents the median frequency of each group. The bottom and top dashed lines represent the upper quartile and lower quartile percentiles, respectively

Because there were significant differences between the OS of the high‐infiltrating groups and that of the low‐infiltrating groups, we speculated that differences in the genome, transcriptome and biological pathways might also exist among different infiltrating groups. The enrichment scores of 50 biological pathways were calculated for MIBC patients in the TCGA‐BLCA cohort, and we found that patients with a low‐infiltrating TME had higher enrichment scores of tumor proliferation‐associated pathways such as the MYC target, the G2M checkpoint, DNA repair, and MTORC1 signaling, and patients with a high‐infiltrating TME had higher enrichment scores of immune‐associated pathways such as the inflammatory response, allograft rejection, epithelial‐mesenchymal transition, and complement (Figure S3A). Several studies have reported that noncoding RNAs, including miRNAs and lncRNAs, play important roles in immune responses.^35^ Therefore, we examined the immune‐related noncoding RNAs of MIBC patients by comparing the transcriptome data of the different infiltrating groups. A total of 6 immune‐related lncRNAs (Figure S3B) and 11 immune‐related miRNAs (Figure S3C) were identified using the limma R package and Venn diagrams. Among the six lncRNAs, the expression levels of AC092580.4, USP30‐AS1, CTA‐384D8.35, and AC002331.1 were the highest in the high‐infiltrating group, followed by the median‐infiltrating group, and lowest in the low‐infiltrating group. The expression levels of GATA3‐AS1 and AC019117.1 were the highest in the high‐infiltrating group, followed by the median‐infiltrating group, and lowest in the low‐infiltrating group. Among the 11 miRNAs, the expression levels of 6 miRNAs (mir142, mir223, mir7702, mir4772, mir155, and mir150) were the highest in the high‐infiltration group, followed by the median‐infiltrating group, and the lowest in low‐infiltrating group, and the expression levels of the remaining 5 miRNAs (mir187, mir429, mir200a, mir551b, and mir200b) were the highest in high‐infiltrating group, followed by the median‐infiltrating group, and the lowest in low‐infiltrating group (Figure 2A). Then, the relationships between the immune‐related noncoding RNAs were analyzed using a correlation analysis, and the results were generally consistent with the above results. Among the six lncRNAs, the expression levels of AC092580.4, USP30‐AS1, CTA‐384D8.35, and AC002331.1 were positively correlated with the abundance of most immune cells and the remaining lncRNAs were negatively correlated with the abundance of most immune cells (Figure S4A). Among the 11 miRNAs, the expression levels of mir142, mir223, mir7702, mir4772, mir155, and mir150 were positively correlated with the abundance of immune cells, and the remaining miRNAs were negatively correlated with the abundance of immune cells (Figure S4B). The mutations and copy number variations (CNVs) of the MIBC genome were then compared among the different infiltrating groups. Twenty‐five common mutated genes of MIBC are shown in Figure 2B. The top five genes in the high‐infiltrating group were TP53 (52%), TTN (48%), KMT2D (33%), ARID1A (32%), and MUC16 (26%), those in the median‐infiltrating group were TTN (51%), TP53 (50%), MUC16 (32%), KMT2D (31%), and KDM6A (26%), and those in the low‐infiltrating group were TP53 (43%), TTN (40%), KMT2D (28%), KDM6A (32%), and ARID1A (27%). Interestingly, we found that the frequencies of CNVs were lower in patients with a high‐infiltrating TME than in patients with a low‐infiltrating TME (Kruskal‐Wallis test, *P* < .001) (Figure 2C,D).

### 3.3 Cluster and functional analyses of DEGs

**Figure 3:**
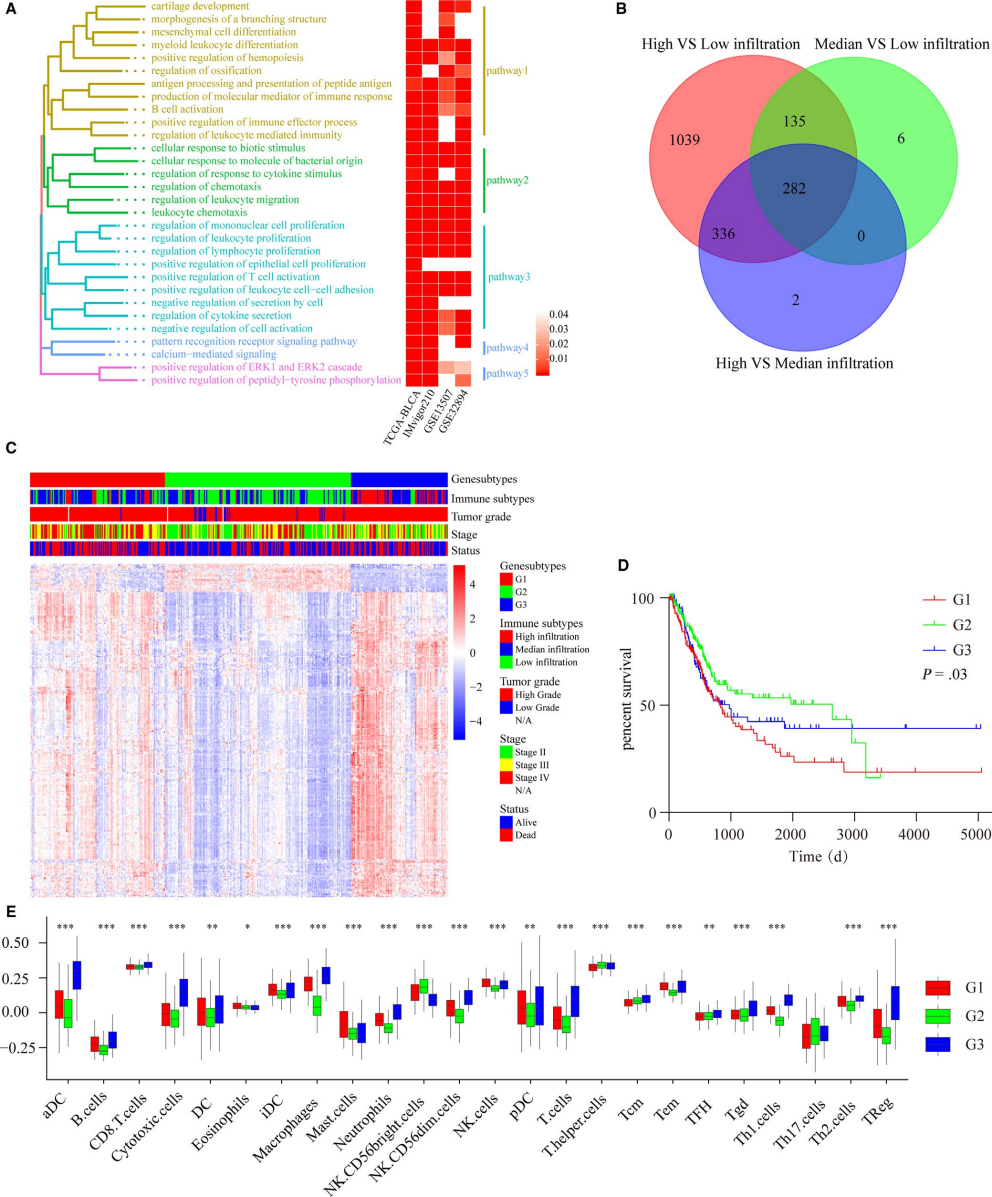
Cluster and functional analyses of DEGs. (A) Common enriched pathways of the DEGs from the four cohorts. Left: tree diagram showing the similarity of the enriched pathways. Right: Heatmap showing the *P* values of the enriched pathways from the four cohorts. (B) Venn diagram showing the number of DEGs among the three immune‐infiltrating subtypes of 384 patients from TCGA‐BLCA. (C) Unsupervised clustering of 282 DEGs from TCGA‐BLCA: red: Gene subtype 1 (G1), green: Gene subtype 2 (G2), blue: Gene subtype 1 (G3). Parameters including immune subtypes, tumor grade, pathological stage, and survival status are shown above the heatmaps. (D) Kaplan‐Meier curves of the overall survival of the three gene subtypes: G1 subtype (red), G2 subtype (green), and G3 subtype (blue). (log‐rank test, *P* = .03). (E) Relative abundances of 24 immune cells in the three gene subtypes: G1 subtype (red), G2 subtype (green), and G3 subtype (blue). The Kruskal–Wallis test was used to compare the differences in immune cells among the three gene subtypes. The boxes represent the 25%‐75% data range, and the whiskers encompass the 95% data range (*P < .05, **P < .01, ***P < .001)

We identified DEGs associated with immune infiltration by comparing the expression levels of mRNAs between the high‐infiltrating groups and the low‐infiltrating groups. A total of 1802, 935, 525, and 371 immune infiltration‐associated DEGs were found in the TCGA‐BLCA cohort, IMvigor210 cohort, http://www.ncbi.nlm.nih.gov/geo/query/acc.cgi?acc=GSE13507 cohort, and http://www.ncbi.nlm.nih.gov/geo/query/acc.cgi?acc=GSE32894 cohort, respectively (Table S1). Pathway enrichment analysis of the DEGs was performed using the GO database, and similar GO terms of the four cohorts were shown in the form of a heatmap and tree diagram. As shown in Figure 3A, multiple immune‐related pathways, such as myeloid leukocyte differentiation, regulation of leukocyte cell‐cell adhesion, regulation of T‐cell activation, positive regulation of lymphocyte proliferation, B‐cell activation, positive regulation of the immune effector process, and regulation of leukocyte‐mediated immunity were analogously enriched in the four cohorts. Then, 282 DEGs were identified by comparing the expression levels of mRNAs across the high‐infiltrating groups, median‐infiltrating groups, and low‐infiltrating groups (Figure 3B). Next, patients were classified into three gene subgroups (G1, G2, and G3) through unsupervised hierarchical clustering of the 282 DEGs (Figure 3C). Afterward, the survival times of the G1, G2, and G3 groups were compared, as shown in Figure 3D. OS was significantly different among the three groups (log‐rank test, *P* = .0278). We then compared the relative quantity of the 24 immune cells among the 3 subgroups. For most immune cells, the relative quantity of immune cells in the G1 group and the G3 group was significantly higher than that in the G2 group (Figure 3E).

### 3.4 Establishment of the TIM signature

**Figure 4:**
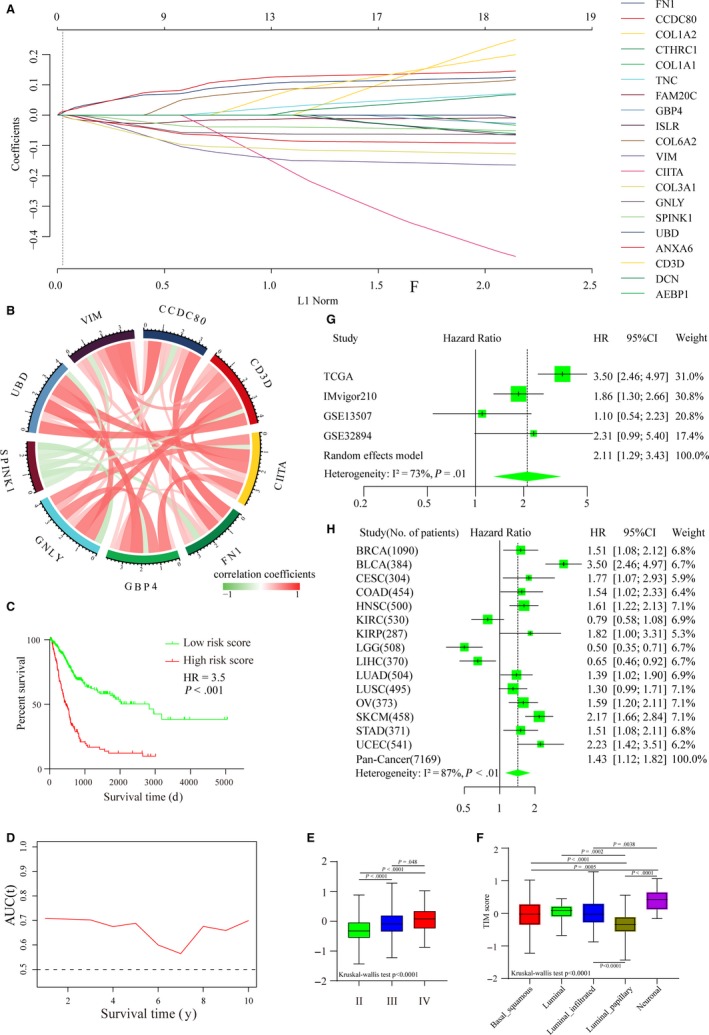
Establishment of the TIM signature. (A) LASSO regression coefficient profiles of 20 core prognostic DEGs (FN1, CCDC80, COL1A2, CTHRC1, COL1A1, TNC, FAM20C, GBP4, ISLR, COL6A2, VIM, CIITA, COL3A1, GNLY, SPINK1, UBD, ANXA6, CD3D, DCN, and AEBP1). (B) Correlation relationships among the nine key prognostic DEGs (CCDC80, CD3D, CIITA, FN1, GBP4, GNLY, SPINK1, UBD, and VIM). (C) Kaplan‐Meier curves of the overall survival of patients with high‐risk scores and patients with low‐risk scores (HR = 3.5, log‐rank test, *P* < .001). (D) The area under the ROC curve (AUC) values of the TIM signature for the prediction of 1 ~ 10‐year OS in TCGA‐BLCA patients. (E) The TIM risk scores of patients with stage II, III, and IV cancer. The boxes represent the 25%‐75% data range, and the whiskers encompass the 95% data range. Statistical differences among the five groups were compared using the Kruskal‐Wallis test. (F) The TIM risk scores of patients according to the five molecular subtypes. The boxes represent the 25%‐75% data range, and the whiskers encompass the 95% data range. Statistical differences among the five groups were compared using the Kruskal‐Wallis test. (G) Forest plot of HRs for patients with high TIM scores vs patients with low TIM scores (pooled HR = 2.11, 95% CI = 1.29‐3.43; I^2^ = 73%, *P* = .01). (H) Forest plot of HRs for 7169 pan‐cancer patients from TCGA with high TIM scores vs patients with low TIM scores from 15 solid tumors with the random effects model (pooled HR = 1.43, 95% CI = 1.12‐1.82; I^2^ = 87%, *P* < .01)

To explore the relationships between the DEGs and the prognosis of MIBC patients, univariate Cox regression analysis was performed with the 282 DEGs using the survival R package. A total of 20 DEGs (FN1, CCDC80, COL1A2, CTHRC1, COL1A1, TNC, FAM20C, GBP4, ISLR, COL6A2, VIM, CIITA, COL3A1, GNLY, SPINK1, UBD, ANXA6, CD3D, DCN, and AEBP1) were found to be highly associated with OS in MIBC patients (*P* < .01). Then, 9 key prognostic genes were selected from the 20 prognostic DEGs using LASSO Cox regression (Figure 4A). The TIM signature, which consisted of the 9 key prognostic genes, was constructed using the respective regression coefficients. The TIM risk scores of MIBC patients were calculated according to the following formula: Risk score = (0.07911684 × expression level of CCDC80) + (−0.053773696 × expression level of CD3D) + (−0.088629069 × expression level of CIITA) + (0.069291126 × expression level of FN1) + (−0.024126876 × expression level of GBP4) + (−0.085878795 × expression level of GNLY) + (−0.04982701 × expression level of SPINK1) + (−0.030284238 × expression level of UBD) + (0.030476199 × expression level of VIM). The relationships among the nine genes are shown in Figure 4B. CCDC80, CD3D, CIITA, FN1, GBP4, GNLY, UBD, and VIM were positively correlated with each other, but SPINK1 was negatively correlated with the other eight genes. Then, OS differences between patients with high TIM risk scores and patients with low TIM risk scores were compared using Kaplan‐Meier curves followed by the log‐rank test, and we found that patients with high TIM risk scores had a worse prognosis than patients with low TIM risk scores (Figure 4C). The AUC values of the TIM signature for the prediction of 1 ~ 10‐year OS are shown in Figure 4D, and the results suggested that the TIM signature had good sensitivity and specificity for prognostic prediction with an AUC of >0.5. Tumor‐node‐metastasis (TNM) classification is an important tool for prognostic prediction, in which a higher stage indicates a worse prognosis. As Figure 4E shows, stage IV MIBC patients had higher TIM risk scores than stage II and stage III MIBC patients. The molecular subtype (basal squamous subtype, luminal subtype, luminal infiltrated subtype, luminal papillary subtype, and neuronal subtype) of MIBC has also been reported to be associated with overall survival, with the luminal papillary subtype having the best survival, while the neuronal subtype has the worst survival.^36^ After comparing the TIM scores of the five molecular subtypes, we found that the luminal papillary subtype had lowest TIM score and the neuronal subtype had the lowest TIM score (Figure 4F). To further validate the stability of the TIM signature in the prognostic prediction of MIBC patients, we pooled the HRs and 95% CIs of the four cohorts using the meta R package, and the result validated that a high TIM risk score was associated with worse prognosis and a low TIM risk score was associated with a better prognosis (HR = 2.11, 95% CI = 1.29‐3.43) (Figure 4G). The HRs of 15 solid tumors, including 7169 pan‐cancer patients, were then pooled to further assess the performance of the TIM signature. Although there was heterogeneity among the HRs of different tumors, the result validated that the TIM risk score could predict that cancer patients with high‐risk scores were associated with a worse prognosis and that cancer patients with low‐risk scores were associated with a better prognosis (HR = 1.43, 95% CI = 1.12‐1.82) (Figure 4H).

### 3.5 The TIM signature was associated with the immune infiltration of the TME

**Figure 5:**
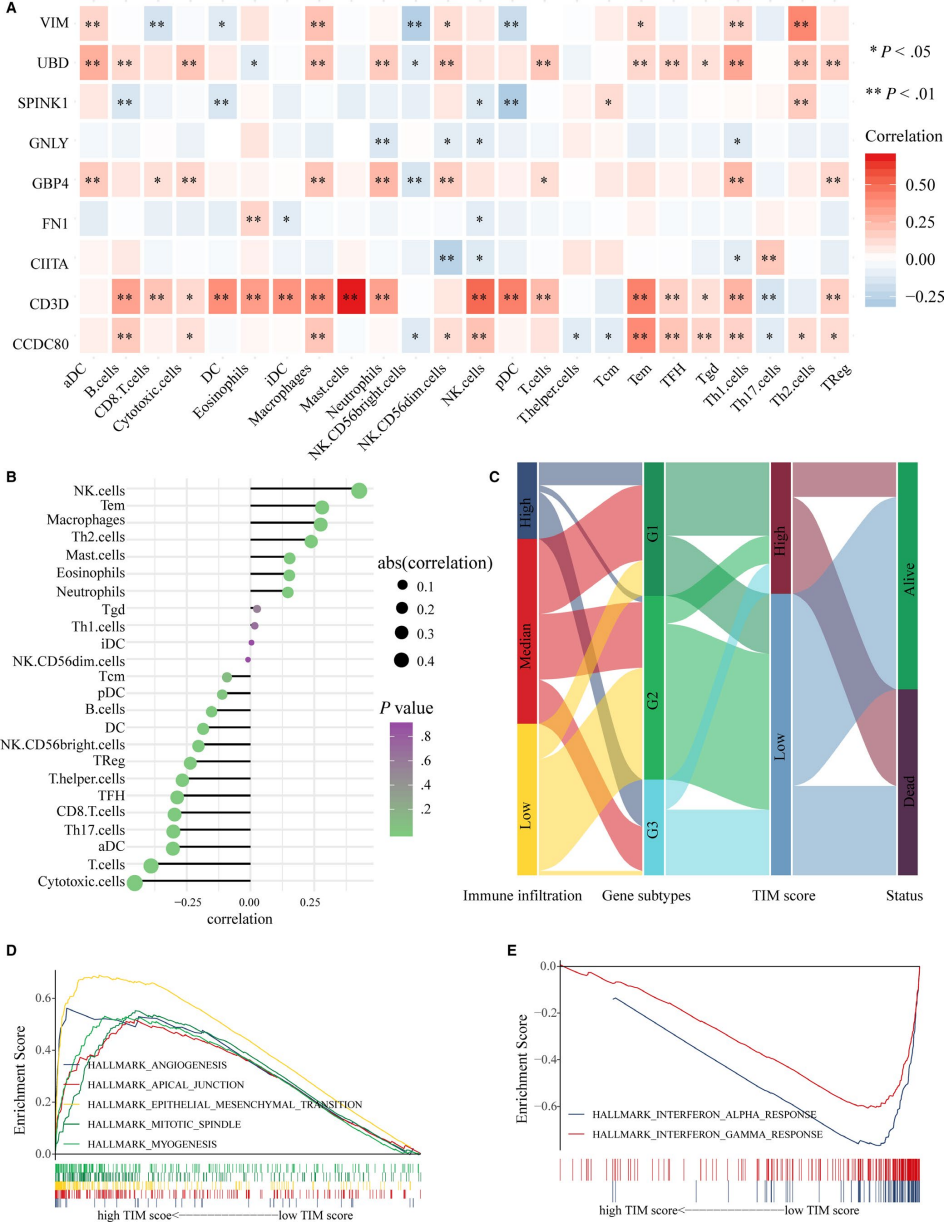
The TIM signature was associated with the immune infiltration of the TME. (A) Correlation between the relative abundances of 24 immune cells and 9 key prognostic DEGs. (*P < .05, **P < .01). (B) Correlation between the relative abundances of 24 immune cells and the TIM risk score. (C) Fractions of MIBC patients were shown in the form of a Sankey map according to different classifications (immune‐infiltrating subtypes: high, median, and low; gene subtypes: G1, G2, and G3; TIM score: high and low; survival status: dead and alive). (D) Enriched pathways of patients with high TIM risk scores using Hallmark gene sets v6.2. (E) Enriched pathways of patients with low TIM risk scores using Hallmark gene sets v6.2

The correlation between the 9 key prognostic DEGs and 24 immune cells is shown in Figure 5A, and we found that the expression levels of UBD, GBP4, CD3D, and CCDC80 were highly related to the abundances of multiple immune cells. Then, correlations between PD‐L1 mRNA and the nine key prognostic DEGs were calculated, and we found that GBP4 (*r* = .64), GNLY (*r* = .69), and UBD (*r* = .63) were highly correlated with PD‐L1 with *r* > .6 (Figure S5). We then analyzed relationships between the TIM risk score and immune cells and found that the TIM risk score was negatively correlated with the abundances of adaptive immunocytes (cytotoxic cells, T cells, aDCs, Th17 cells, and so on), and positively correlated with the abundances of innate immunocytes (NK cells, macrophages, mast cells, and so on) (Figure 5B). A Sankey map was depicted to show the relationships between the TIM score and tumor subtypes, and the result suggested that patients with low TIM scores were mainly linked to the high immune‐infiltrating gene subtypes (G3) and better survival status (Figure 5C). GSEA was then performed to test whether the genes of patients with high TIM scores or low TIM scores were enriched in previously defined biological pathways. We found that the genes of patients with a high TIM score were enriched in protumor‐associated pathways, including angiogenesis, apical junction, epithelial‐mesenchymal transition, mitotic spindle, and myogenesis (Figure 5D), and the genes of patients with a low TIM score were enriched in immune‐associated pathways, including interferon alpha response and interferon gamma response (Figure 5E). In conclusion, the TIM signature might have potential value for inferring the TME characteristics of MIBC patients.

### 3.6 The TIM signature could predict the immunotherapeutic response of MIBC patients

**Figure 6:**
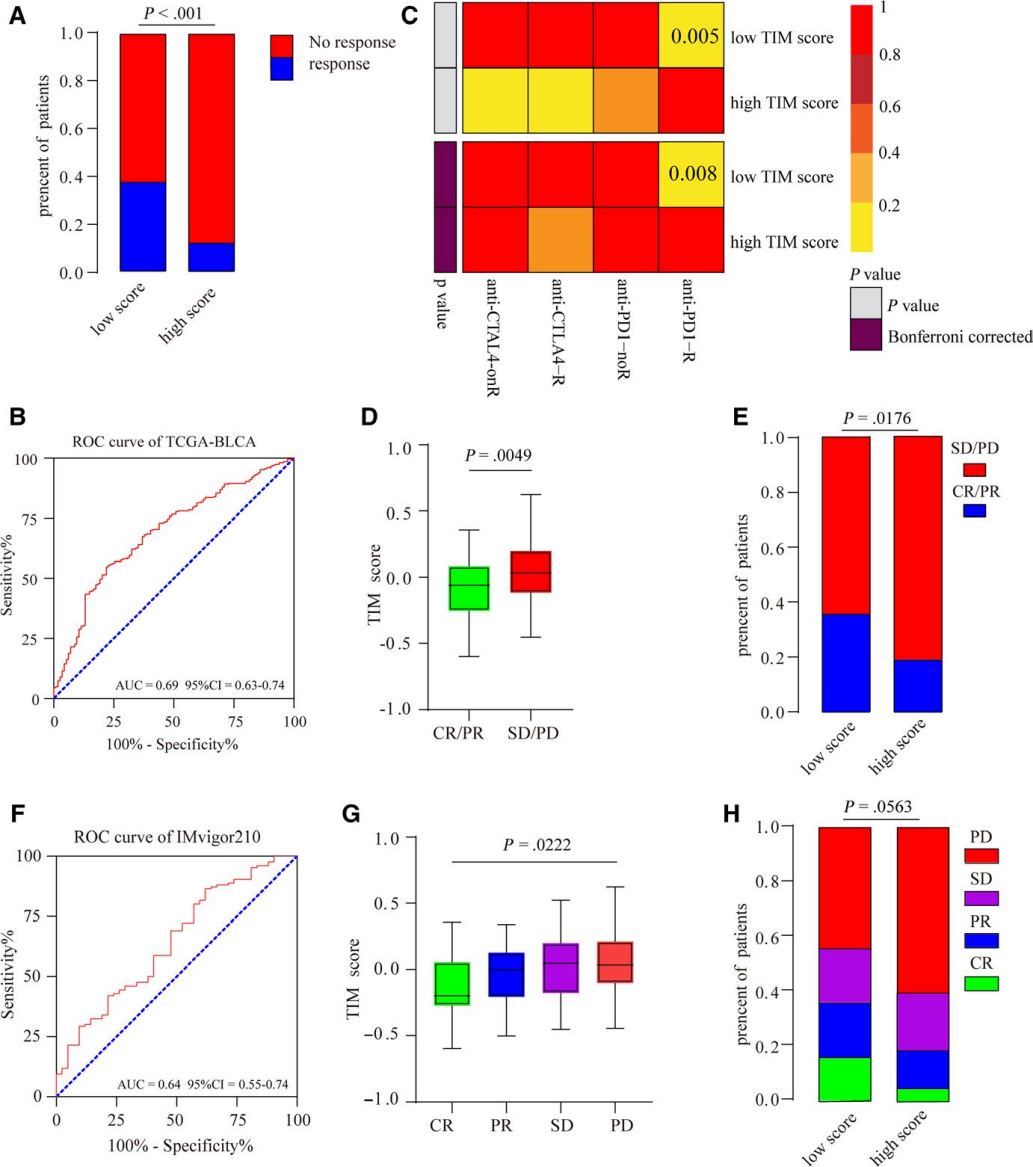
The TIM signature could predict the immunotherapeutic response of MIBC patients. (A) Rates of the different anti‐PD1 and anti‐CTLA4 responses of patients from the TCGA‐BLCA cohort predicted by the Tumor Immune Dysfunction and Exclusion (TIDE) web program in the high or low TIM score groups (Chi‐square test, *P* < .001). (B) Receiver operating characteristic (ROC) curves evaluating accuracy of the TIM signature for predicting the anti‐PD1 and anti‐CTLA4 response of patients from the TCGA‐BLCA cohort (AUC (area under the ROC curve)=0.69, 95% CI (confidence interval)=0.63‐0.74). (C) Submap analysis revealed that patients with low TIM scores are more responsive to anti‐PD1 treatment (Fisher's exact test, *P* = .005, Bonferroni‐corrected *P* = .008). (D) The TIM scores of patients from the IMvigor210 cohort with different anti‐PD‐L1 response statuses (stable disease (SD)/progressive disease (PD), complete response (CR)/partial response (PR)). The bottom and top of the boxes represent the upper quartile and lower quartile percentiles, respectively. The whiskers encompass the maximum and minimum expression levels (Mann‐Whitney U test, *P* = .0049). (E) The rates of different anti‐PD‐L1 responses of patients from the IMvigor210 cohort in the high or low TIM score groups (SD/PD, CR/PR) (chi‐square test, *P* = .0176). (F) ROC curves evaluating the accuracy of the TIM signature for predicting the anti‐PD‐L1 response of patients from the IMvigor210 cohort (AUC = 0.64, 95% CI = 0.55‐0.74). (G) The TIM scores of patients from the IMvigor210 cohort with different anti‐PD‐L1 response statuses (SD, PD, CR, and PR). The bottom and top of the boxes represent the upper quartile and lower quartile percentiles, respectively. The whiskers encompass the maximum and minimum expression levels (Kruskal‐Wallis test, *P* = .0222). (H) The rates of different anti‐PD‐L1 responses of patients from the IMvigor210 cohort in the high or low TIM score groups (SD, PD, CR, and PR) (Chi‐square test, *P* = .0563)

Because the TIM score was highly associated with the prognosis of MIBC patients and density of immune infiltration, we then tested whether the TIM signature could predict the immunotherapeutic response of ICIs. The TIDE web program was used to infer the immunotherapeutic response of TCGA‐BLCA patients, and we excitedly found that patients with a low TIM score had a higher response rate than patients with a high‐risk score (Chi‐square test, *P* < .001) (Figure 6A). To validate our finding, subclass mapping was used to compare the similarity between the expression profiles of the TCGA‐BLCA samples and an immunotherapy‐response melanoma cohort. The results showed that patients with a low TIM score more likely responded PD‐1 inhibitor immunotherapy (Bonferroni‐corrected *P* = .008) (Figure 6C). The predictive ability of the TIM score was also confirmed in the IMvigor210 cohort, which had detailed immunotherapy information. Patients who responded to immunotherapy had low TIM scores (Figure 6D,G), and patients with low TIM scores had higher response rates than patients with high‐risk scores (Figure 6E,H). Finally, ROC cures showed that the TIM signature was an ideal model for predicting the immunotherapeutic response of MIBC patients (Figure 6B: AUC = 0.69, 95% CI = 0.63‐0.74; Figure 6F: AUC = 0.64, 95%=0.55‐0.74).

## 4 DISCUSSION

Immunotherapies are emerging treatment options for MIBC patients, but immunotherapeutic responses have been heterogeneous among MIBC patients.^37^ Moreover, immunotherapies are expensive for patients, so the accurate prediction of immunotherapeutic responses will be helpful to save medical costs for insensitive patients. Previous studies have shown that the reactivation of the antitumor activities of immune cells is necessary for the effectiveness of immunotherapies, especially ICIs.^12^, ^13^ However, as mentioned before, the TME includes many types of immune cells, which have complex biological relationships with tumor cells and could lead tumors toward either progression or repression. Therefore, we speculated that the diversity of immune cells in TME might be one of the causes that contributes to differences in the immunotherapeutic response. In this study, we estimated the relative quantity of 24 immune cells based on transcriptome data from MIBC patients, and unsupervised hierarchical clustering analysis showed that immune infiltrations were indeed heterogeneous among MIBC patients. Survival analysis showed that prognosis was significantly different between patients with a high‐infiltrating TME and patients with a low‐infiltrating TME. To simplify the relationship between the TME and the prognosis of MIBC patients, we first constructed a gene signature, the TIM signature, which consists of nine prognostic‐relevant DEGs for predicting the prognosis of MIBC patients. We also found that the immunotherapeutic response rate of patients with low TIM scores was significantly higher than that of patients with high TIM scores. ROC analysis validated that the TIM signature had an ideal ability for predicting the immunotherapeutic responses of MIBC patients.

TME‐relevant DEGs were identified by comparing the expression profiles of patients with a high‐infiltrating TME and that of patients with a low‐infiltrating TME. In addition, GO analysis showed that the TME‐relevant DEGs of the four cohorts were collectively enriched in immune‐relevant biological pathways, such as immune cell activation and proliferation. Moreover, the enrichment scores of 50 biological pathways were calculated for MIBC patients. By comparing enrichment scores between patients with a high‐infiltrating TME and patients with a low‐infiltrating TME, we observed that cell proliferation‐relevant pathways were enriched in tumors with a low‐infiltrating TME, and immune‐relevant pathways were enriched in tumors with a high‐infiltrating TME. These results suggest that high‐infiltrating TMEs might have a stronger immune response, which contributes to the repression of tumors. We also observed that the expression levels of PD‐L1 in the high‐infiltrating TME groups were higher than those of the low‐infiltrating TME groups, and we inferred that high PD‐L1 expression might help tumor cells of high‐infiltrating TMEs to tolerate the antitumor activities of immune cells. This finding suggested that ICIs such as PD‐L1 blockers would be effective for patients with high immune infiltration, who had stronger immunological surveillance. Similarly, several studies have reported that tumor patients with high immune infiltration had a better prognosis and a higher immunotherapeutic response rate.^16^, ^38^, ^39^, ^40^ Overall, the rapid assessment of the immune infiltration of the TME could be a way to predict the immunotherapeutic response of MIBC patients.

Nine key prognostic genes (CCDC80, CD3D, CIITA, FN1, GBP4, GNLY, SPINK1, UBD, and VIM) were included in the TIM signature. Correlation analysis showed that UBD, GBP4, CD3D, and CCDC80 were highly related to the abundance of immune cells. Several studies have observed that some of these genes play important roles in the regulation of the immune response. For example, CIITA can trigger antitumor immunity by inducing the expression of the MHC class II molecules of tumor cells,^41^ CD3D can regulate the proliferation and development of T cells,^42^, ^43^ and GNLY can encode granulysin, which can cause the lysis of tumor cells.^44^ Interestingly, using the median TIM score as a cutoff value, we observed that the genes of patients with high TIM scores were enriched in protumor pathways and that the genes of patients with low TIM scores were enriched in inflammatory pathways. Sankey maps showed that patients with low TIM scores were mainly linked to the high immune‐infiltrating gene subtypes and better clinical outcomes. The results suggested that the TIM signature was highly associated with the immune microenvironment and could identify patients with a high‐infiltrating TME from patients with a low‐infiltrating TME.

By comparing the noncoding RNAs of different immune‐infiltrating groups, we found that 6 differentially expressed lncRNAs (AC092580.4, CTA‐384D8.35, AC002331.1, USP30‐AS1, GATA3‐AS1, and AC019117.1) and 11 differentially expressed miRNAs (mir142, mir223, mir7702, mir4772, mir155, mir150, mir187, mir429, mir200a, mir551b, and mir200b) were associated with immune infiltration. Interestingly, several previous studies similarly reported that some of these noncoding RNAs were involved in the regulation of the immune response. For example, Gibbons et al reported that GATA3‐AS1 was specifically expressed in Th2 cells and could induce the expression of GATA3 in Th2 cells by remodeling the chromatin of the GATA3‐AS1‐GATA3 locus.^45^, ^46^ As another example, mir142, also called hematopoietic‐specific miRNA, plays important role in the regulation of the immune system. Sun et al reported that mir142 could interact with atypical E2F transcription factors to control T‐cell proliferation in graft‐vs‐host disease (GVHD) models,^47^ and Berrien‐Elliott et al found that the survival of cytotoxic type 1 innate lymphoid cells was dependent on the abundant expression of mir142. Therefore, it is not surprising that mir142 was highly expressed in highly infiltrating TMEs.^48^ Moreover, miR‐223,^49^ mir155,^50^, ^51^ mir187,^52^ and mir200b^53^ have also been reported to be associated with the immune response, so it will be worthwhile to conduct more research exploring the relationships between noncoding RNAs and the immune response in the future.

The genetic alterations of MIBC, including mutations and CNVs, were compared in this study. Genetic mutations could generate alterations in the amino acids of proteins, and these abnormal proteins of tumor cells, called neoantigens, could stimulate the adaptive immune response and enhance the checkpoint inhibitor response.^54^ A previous study reported that the frequencies of mutations were high in MIBC genomics, and approximately 68% of MIBC patients had genomic alterations; the high frequencies of mutations might be one reason why ICIs were effective for MIBC patients.^55^ In this study, we observed that the mutation frequencies of common mutated genes such as TP53, TTN, and KMT2D were slightly higher in high‐infiltrating TMEs than in low‐infiltratingTMEs. But the frequencies of mutations in MIBC patients are overall high, it is not surprising that the differences were not obvious among patients with different infiltrating TMEs. CNVs are duplications or deletions of continuous base pairs of genes, and several studies have found that higher frequencies of CNVs always predict a worse prognosis in tumor patients.^56^, ^57^ CNVs were also found to be associated with resistance to ICIs such as anti‐CTLA‐4 therapy in melanoma.^54^ Our study also found that the frequencies of genomic CNVs were higher in low‐infiltrating TMEs than in high‐infiltrating TMEs. The results suggested that CNVs might be important predictors for the prognosis and immunotherapeutic response of MIBC patients.

Our study provided an ideal predictor for the prognosis and immunotherapeutic response of MIBC patients, but our study still had some limitations. First, additional prognostic predictors, such as clinicopathological characteristics, were not evaluated in this study, and the signature will be more accurate if these parameters are included. Second, we estimated the relative quantity of immune cells using transcriptome data from MIBC patients. Other methods, such as immunohistochemistry and flow cytometry, will be helpful to validate our results in the future. Third, the stability of the TIM signature was tested through the cross validation of four MIBC cohorts, but we think the signature will be more reliable if it is tested by prospective cohort studies in the future. However, the TIM signature simplified the complicated relationships between immune infiltration and clinical features, including survival outcomes and immunotherapeutic responses, and made it easy to predict prognosis and immunotherapeutic response in MIBC patients. At the same time, we compared the genome and transcriptome of patients with different infiltrating TMEs and provided several novel biomarkers for clinical diagnosis and drug research.

## 5 CONCLUSIONS

In conclusion, our study depicted the landscape of the TME in MIBC patients and found that the genome and transcriptome were heterogeneous among patients with different infiltrating TMEs. Moreover, we constructed an immune‐relevant gene signature that had a good ability to predict prognosis and immunotherapeutic responses in MIBC patients.

## CONFLICT OF INTEREST

The authors declare no conflicts of interest.

## Data Availability

The datasets supporting the conclusions of this article were downloaded from The Cancer Genome Atlas (TCGA, https://tcga-data.nci.nih.gov/tcga/) and Gene Expression Omnibus (GEO, http://www.ncbi.nlm.nih.gov/geo/).
