# Supplementary figures and images for: An immune relevant signature for predicting prognoses and immunotherapeutic responses in patients with muscle‐invasive bladder cancer (MIBC)
Source: Cancer Med. 2020 Feb 25;9(8):2774–90. doi: 10.1002/cam4.2942 (PMC7163112; doi:10.1002/cam4.2942)

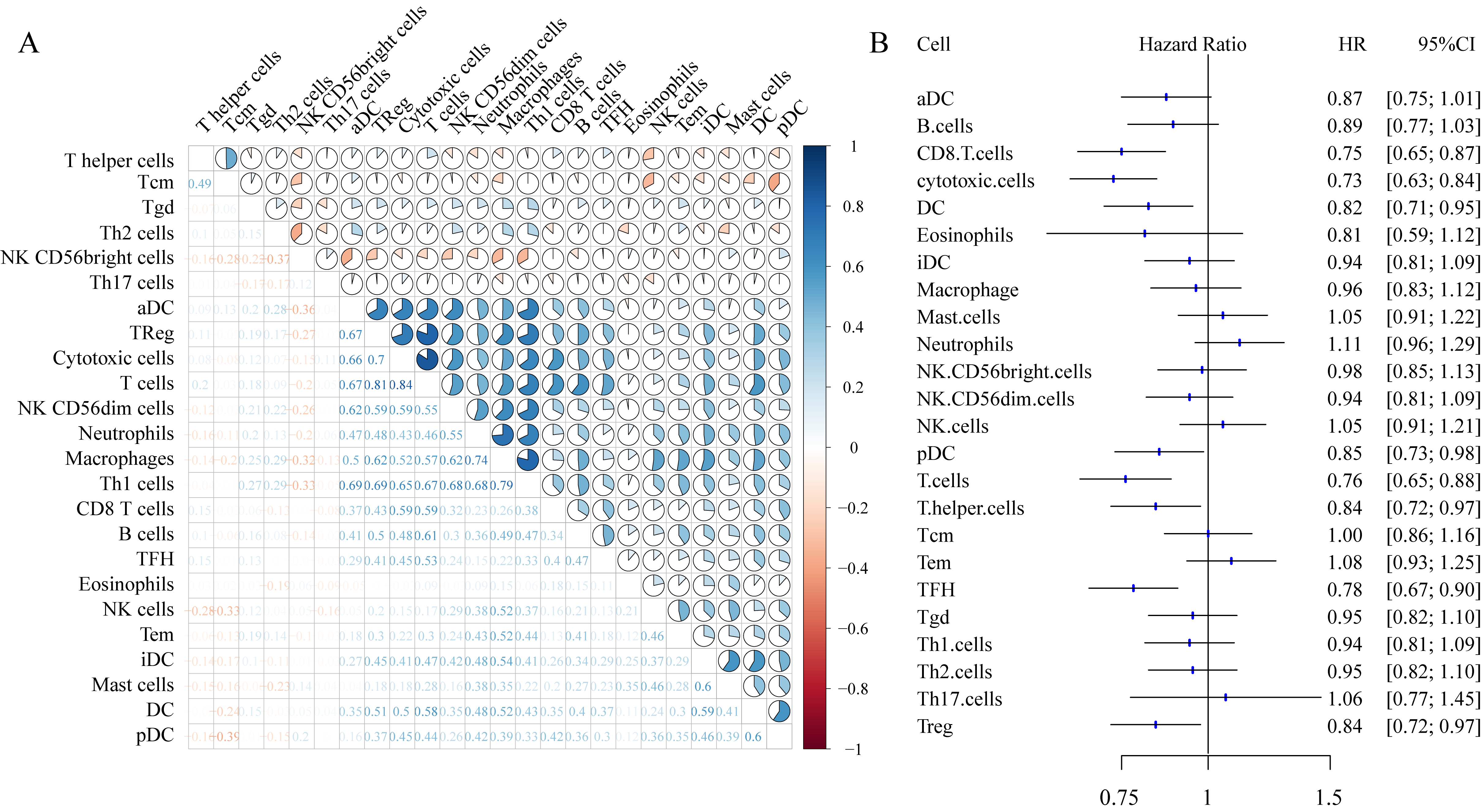

Supplement: Supplementary file 1 [file CAM4-9-2774-s001.tif]

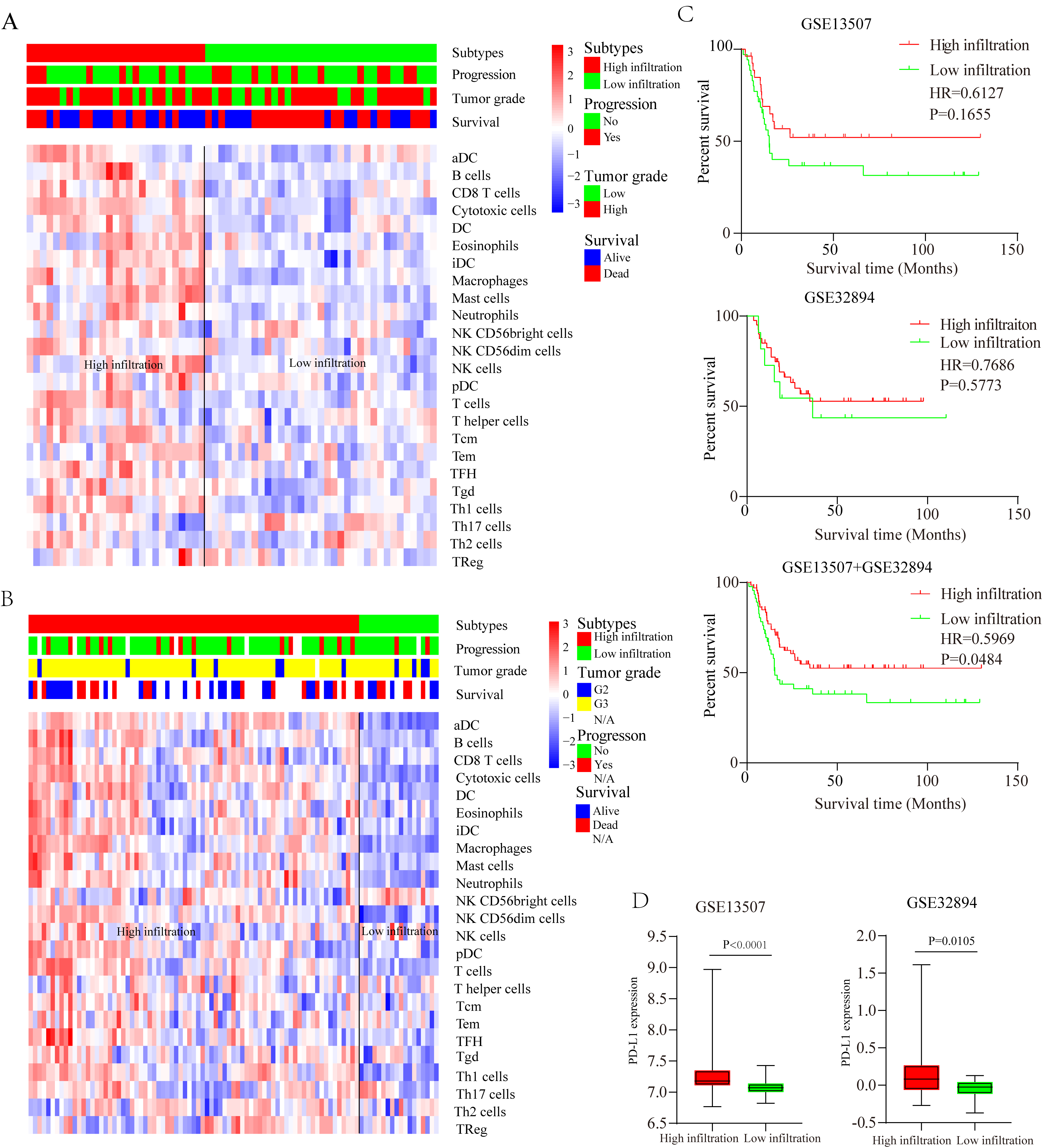

Supplement: Supplementary file 2 [file CAM4-9-2774-s002.tif]

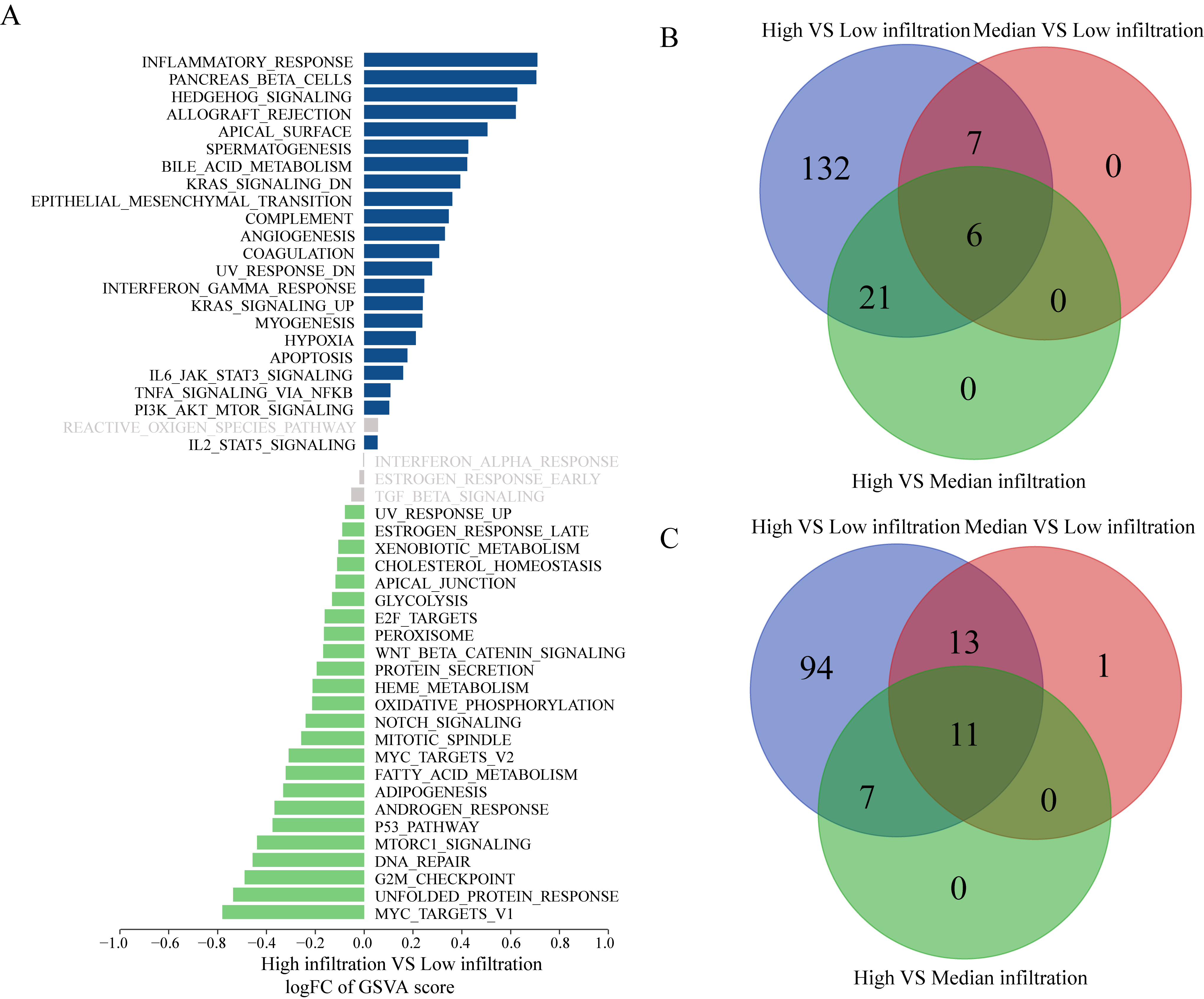

Supplement: Supplementary file 3 [file CAM4-9-2774-s003.tif]

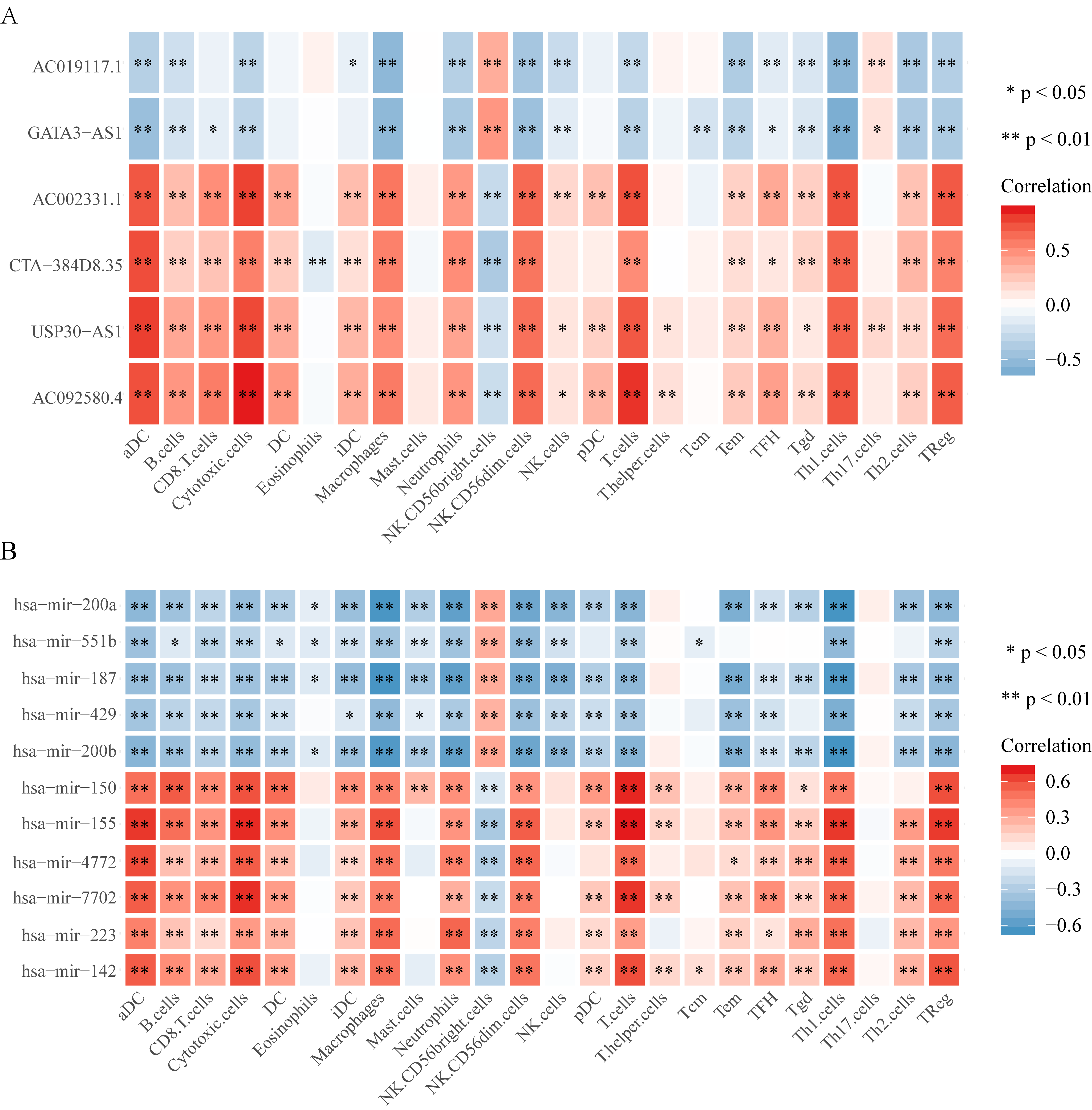

Supplement: Supplementary file 4 [file CAM4-9-2774-s004.tif]

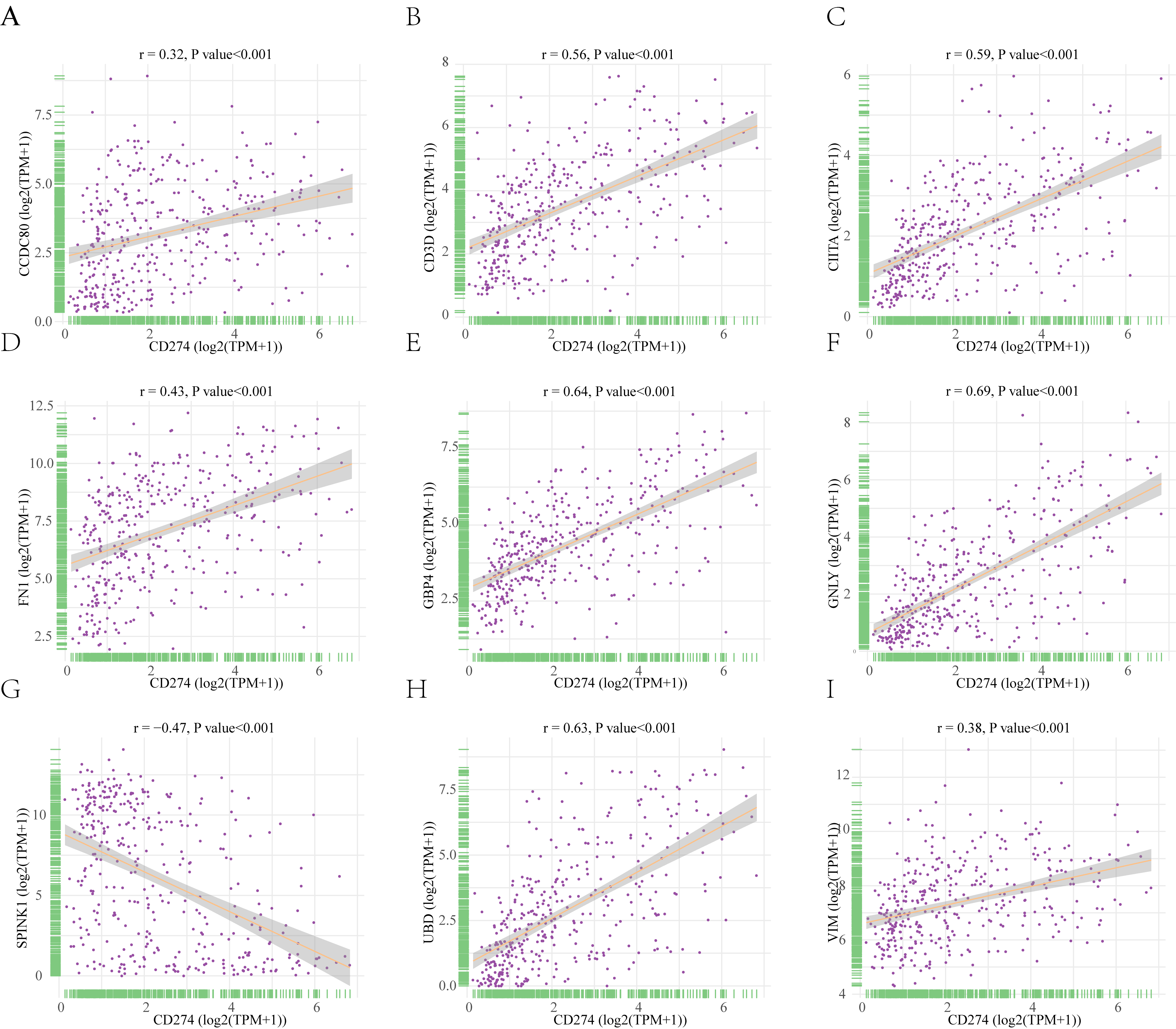

Supplement: Supplementary file 5 [file CAM4-9-2774-s005.tif]
